# Dermal fibroblasts induce cell cycle arrest and block epithelial–mesenchymal transition to inhibit the early stage melanoma development

**DOI:** 10.1002/cam4.707

**Published:** 2016-04-06

**Authors:** Linli Zhou, Kun Yang, R. Randall Wickett, Yuhang Zhang

**Affiliations:** ^1^ Division of Pharmaceutical SciencesCollege of PharmacyUniversity of CincinnatiCincinnatiOhio45267

**Keywords:** Melanoma, stromal fibroblasts, tumor microenvironment, Wnt signaling, *β*‐catenin

## Abstract

Stromal fibroblasts are an integral part of the tumor stroma and constantly interact with cancer cells to promote their initiation and progression. However, the role and function of dermal fibroblasts during the early stage of melanoma development remain poorly understood. We, therefore, designed a novel genetic approach to deactivate stromal fibroblasts at the onset of melanoma formation by targeted ablation of *β*‐catenin. To our surprise, melanoma tumors formed from *β*‐catenin‐deficient group (B16F10 mixed with *β*‐catenin‐deficient fibroblasts) appeared earlier than tumors formed from control group (B16F10 mixed with normal dermal fibroblasts). At the end point when tumors were collected, mutant tumors were bigger and heavier than control tumors. Further analysis showed that there were fewer amounts of stromal fibroblasts and myofibroblasts inside mutant tumor stroma. Melanoma tumors from control group showed reduced proliferation, down‐regulated expression of cyclin D1 and increased expression of cyclin‐dependent kinase inhibitor p16, suggesting dermal fibroblasts blocked the onset of melanoma tumor formation by inducing a cell cycle arrest in B16F10 melanoma cells. Furthermore, we discovered that dermal fibroblasts prevented epithelial‐mesenchymal transition in melanoma cells. Overall, our findings demonstrated that dermal fibroblasts crosstalk with melanoma cells to regulate *in vivo* tumor development via multiple mechanisms*,* and the outcomes of their reciprocal interactions depend on activation states of stromal fibroblasts and stages of melanoma development.

## Introduction

Cutaneous melanoma consists of not only melanoma cells but also a heterogeneous mix of genetically stable non‐cancer cells, including endothelial cells, immune cells, and fibroblasts, embedded in a tumor‐specific extracellular matrix (ECM) 1. It is well accepted that reciprocal interaction between tumor cells and stromal cells contributes to the transformation process by encouraging melanoma growth, angiogenesis, inflammation, and metastasis 2. Infiltrated and surrounding stromal fibroblasts are stimulated into an activated state through continuous paracrine cell–cell communication with tumor cells and transdifferentiate into cancer‐associated fibroblasts (CAFs) 3. CAFs subsequently acquire properties of myofibroblasts and produce various growth factors, cytokines, and ECM proteins, which remodel the stromal architecture of the diseased tissue and create an optimal niche for tumor cells to grow, migrate, and evade death. Therefore, co‐targeting melanoma cells and the host microenvironment including CAFs could potentially provide novel therapeutic approaches 4.

One major source of CAFs in melanoma is resident normal skin fibroblasts (NFs), which are recruited and activated by chemical factors released from melanoma cells 5. CAFs exhibit both phenotypical and physiological differences from NFs. Particularly, it has been reported that CAFs and NFs have opposite effects on tumor cells growth 6. CAFs promote melanoma progression by providing necessary growth factors and remodeling the ECM to facilitate growth and migration of melanoma cells 7. In contrast to CAFs, it appears that NFs suppress growth and progression of premalignant lesions at early stages of tumor development 8. There are several possible causes of this inhibitory effect, including (1) the physical barrier; (2) secretion of cytokines to immobilize immune cells and (3) production of tumor inhibitory proteins. Flach et al. showed that chemical factors secreted by melanoma cells induced fibroblasts to migrate toward, surround, and then infiltrate the tumor mass 9. These signals also stimulate fibroblasts to produce ECM proteins and a pool of pro‐tumorigenic soluble factors. However, the exact mechanisms of how NFs interact with malignant melanoma cells at the initiation stage and subsequently transform to CAFs, which finally contribute to the tumor development, are poorly understood.

Wnt/*β*‐catenin signaling is one of the key signaling pathways that are deregulated in a variety of human cancer cells, including stromal fibroblasts 10. Aberrant activation of *β*‐catenin signaling in fibroblasts leads to diseases such as skin fibrosis 11. Dermal fibroblasts are also actively involved in cutaneous wound healing process, including reconstituting the dermis, secreting ECM molecules, regulating cell adhesion and promoting wound contraction 12. It has been reported that *β*‐catenin not only functions as a transcriptional coactivator to regulate the proliferation and differentiation of fibroblasts, and also constitutes as a critical component of *β*‐catenin/E‐cadherin complex to control fibroblast motility and migration, which greatly influence the wound healing speed and wound size 13.

In this study, we developed a novel genetic approach to deactivate stromal fibroblasts *in vivo* by targeted ablation of *β*‐catenin. Our results demonstrated that dermal fibroblasts function as physical and signaling barriers to block melanoma formation at the initiation stage before melanoma cells stimulate them to transdifferentiate into CAFs.

## Material and Methods

### Mice

*Col1α2‐CreER* mice were generated as described previously 19. C57BL/6J (B6) mice and *Ctnnb1* ^*fl/fl*^ mice were obtained from the Jackson laboratory (Bar Harbor, ME). *Ctnnb1* ^*fl/fl*^ mice were crossed with *Col1α2‐CreER* mice for several generations to generate *Ctnnb1* ^*fl/fl*^ *; Col1α2‐CreER* mice. *Ctnnb1* ^*fl/fl*^ *; Col1α2‐CreER* mice were further back crossed with B6 mice for ten generations. Mice were genotyped by polymerase chain reaction (PCR) analysis of genomic DNA extracted from tail biopsies. The *β*‐catenin alleles were genotyped with the following primer pair: Forward: 5′‐AAGGTAGAGTGATGAAGGTTGTT‐3′ and Reverse: 5′‐CACCATGTCCTCTGTCTA‐3′. The presence of Cre transgene was identified by PCR using the following primer pair: Forward: 5′‐CGGTCTGGCAGTAAAAACTAT‐3′ and Reverse: 5′‐CAGGGTGTTATAAGCAATCCC‐3′. All mice were housed in the Laboratory Animal Services Facility of the University of Cincinnati under an artificial 12/12 light–dark cycle and were allowed free access to normal mouse feedings and water. The Institutional Animal Care and Use Committee of the University of Cincinnati approved all experimental procedures involving mice.

To induce Cre activity, tamoxifen (Sigma‐Aldrich, St. Louis, MO) in corn oil (10 mg/ml) was administered to adult mice by intraperitoneal (i.p.) injection at 1 mg/g body weight for 7 consecutive days. 100 *μ*L bleomycin sulfate (Sigma‐Aldrich) was injected subcutaneously into each mouse every day for 30 days to induce the activation of fibroblasts as described in the text. Back skin of induced mice was harvested at postnatal day 60 (P60) for histological analysis.

### Cells and cell culture

Fibroblast cultures were established according to published protocol 14 . Briefly, dorsal skin biopsies were harvested from 2 or 3‐day‐old *Ctnnb1fl/fl*;* Col1a2‐CreER* mice and control littermates of genotype *Ctnnb1+/fl*;* Col1a2‐CreER*. They were then washed in phosphate buffered saline (PBS) twice, minced with curved scissors into small pieces, and incubated with 5 mg/ml collagenase I for 30 minutes under stirring. Digested skin tissue was further washed twice with calcium‐free Dulbecco's Modified Eagle's Medium (DMEM) supplemented with 10% fetal bovine serum (FBS). After washing, skin pieces were cultured in DMEM supplemented with 10% FBS, 1% MEM nonessential amino acid, 1% Hank's balanced salt solution (HBSS), and 1% Penicillin/Streptomycin (PS) at 37°C with 5% CO_2_ to let fibroblasts crawl out of the tissue fragments and attach to the plate. After 2 passages, the purity of dermal fibroblasts was examined by flow cytometry analysis. Fibroblasts were then seeded at a concentration of 3x10^4^ cells/m for further analysis. *β*‐catenin null mutation in fibroblasts were induced by adding 100 ng/mL 4‐hydroxytamoxifen (4‐OHT) into the culture medium and incubated for 48 h before each experiment. Murine B16F10 melanoma cell line was obtained from American Type Culture Collection (ATCC, Manassas, VA). Cells were maintained in DMEM supplemented with 10% FBS and 1% PS. All cells were incubated at 37°C in a 5% CO_2_, 95% air‐humidified incubator. All cell culture media were purchased from Invitrogen (Invitrogen, Carlsbad, CA) unless otherwise indicated.

### Cell proliferation and cell cycle analysis

*Ctnnb1* ^*fl/fl*^ *, Col1a2‐CreER* fibroblasts and control *Ctnnb1* ^*+/fl*^ *, Col1a2‐CreER* fibroblasts were seeded at 300,000 cells/dish in a 10 cm tissue culture dish. 4‐OHT was then added to culture medium to induce Cre recombinase expression and the ablation of *β*‐catenin. After 48‐h incubation, fibroblasts were disassociated with 0.5% trypsin‐EDTA (Invitrogen) and collected by centrifuge. Cell numbers were counted manually under light‐phase microscope and reseeded for further experiments. To perform cell cycle analysis, cells were washed with ice‐cold PBS and fixed with ice‐cold 70% ethanol for 24 h. After fixation, ethanol was removed by centrifugation, and cells were suspended in 0.5 mL propidium iodide (PI)/RNase solution and incubated at 37°C for 30 min in the dark. Stained cells were analyzed using a BD FACSCanto flow cytometer (Becton Dickinson, San Jose, CA) to quantify DNA content in cells. Cell cycle histograms were generated from flow cytometry data, and the percentage of fibroblasts in each cell cycle phase was determined. Data analysis was performed using the Modfit LT software (Verity Software, Topsham, ME).

### Quantitative real‐time PCR

Total RNA was isolated from mouse dermis using RNeasy Fibrous Tissue mini kit or from cultured fibroblasts using RNeasy Mini Kit from Qiagen (Qiagen, Valencia, CA). Subsequently total RNA was reverse transcribed to complementary DNA (cDNA) using Superscript III kit (Invitrogen). Quantitative PCR (qPCR) reactions were performed using the Power SYBR green dye in the StepOnePlus^™^ Real‐Time PCR system (Applied Biosystems, Foster City, CA) as previously published. qPCR primers for Egf, Fn1, Hgf, MMP2, Tgf*α*, Tgf*β*1, TNC, were purchased from Qiagen. All qPCR data were normalized to GAPDH expression.

### Mouse melanoma tumor grafting model

All animal experiments were conducted with approval from the University of Cincinnati Institutional Animal Care and Use Committee. To induce melanoma formation, B16F10 murine melanoma cells mixed with either mutant *Ctnnb1* ^*fl/fl*^ *; Col1α2‐CreER* fibroblasts or control *Ctnnb1* ^*+/fl*^ *; Col1α2‐CreER* fibroblasts at a ratio of 1:2 were injected intradermally into the flanks of B6 wild‐type mice with a total cell number of 6 × 10^4^ for each tumor. Size of tumors was measured and recorded every other day for a maximum of 7 days after tumor appeared. All mice were then sacrificed, and the tumors were harvested and fixed with 4% (w/w) formaldehyde in PBS for paraffin sections or prepared for western blot analysis.

### Histology and immunohistochemistry

Paraffin or frozen tumor tissue sections were prepared for collagen sirius red staining, histological analysis, BrdU incorporation assays, TUNEL assays, senescence‐associated‐beta‐galactosidease (SA‐*β*‐gal) staining and immunostaining according to published protocols 15, 16, 17. The following primary antibodies were used: anti‐TE‐7 (Millipore, Billerica, MA, USA, 1:200), anti‐*β*‐catenin (Invitrogen, Carlsbad, CA, USA, 15B8, 1:1000), anti‐PDGFR‐α (R&D, Minneapolis, MN, USA, 1:40), anti‐prolyl‐4‐hydroxylase‐ *β*(P4HB) (Abcam, Cambridge, MA, USA, 1:200), anti‐BrdU (Abcam, BU1/75, 1:25), anti‐Ki67 (Imgenex, Littleton, CO, USA, 1:50), anti‐Cyclin D1 (Cell Signaling, Danvers, MA, USA, 1:50), anti‐E‐cadherin (cell signaling, 24E10, 1:400), anti‐Vimentin (Abcam, 1:200), anti‐N‐cadherin (Abcam, 1:200), anti‐phospho‐pRb (cell signaling, 1:200). The images were taken under a Nikon Eclipse 80i fluorescence microscope.

### Immunoblotting

Immunoblotting was performed as described elsewhere. The following primary antibodies were used: anti‐*β*‐catenin (Invitrogen, 15B8, 1:1000), anti‐pERK1/2 MAPK (Cell Signaling, 1:2000), anti‐pospho‐pERK1/2MAPK (Cell Signaling, 1:2000), anti‐pRb (Abcam, 1:1000), and anti‐p16 (Millipore, 1:1000). To verify equal loading of samples, the membranes were incubated with monoclonal *β*‐actin antibody, followed by an HRP‐conjugated goat anti‐mouse IgG.

### Statistical analysis

Statistical analysis of difference was carried out by student's *t*‐test. Data were analyzed using a GraphPad Prism 5.01 software package (GraphPad Software Inc., San Diego, CA) and expressed as the mean ± SEM. Differences are considered significant at *P* < 0.05.

## Results

### Nuclear and cytoplasmic *β*‐catenin are detected in stromal fibroblasts in human melanoma tissue

**Figure 1:**
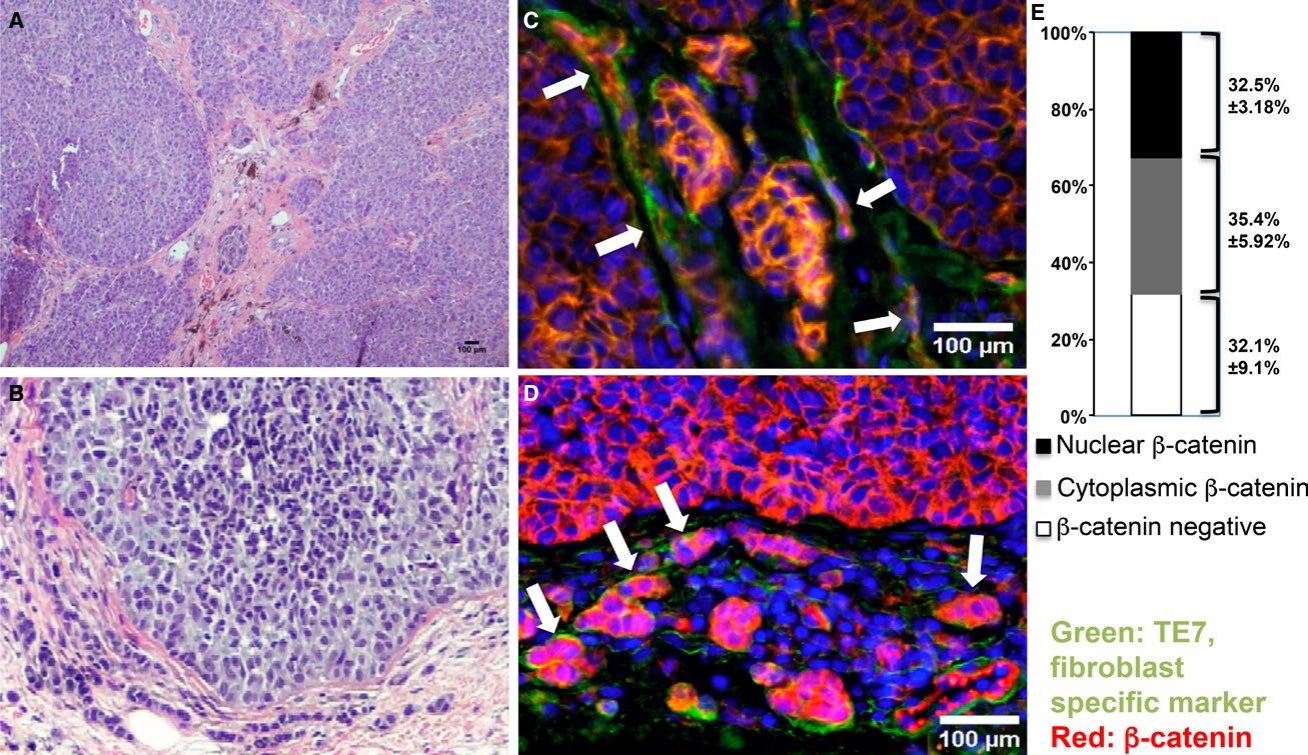
*β*‐catenin is highly expressed in melanoma‐associated fibroblasts. Expression of nuclear and cytoplasmic *β*‐catenin (red) in stromal fibroblasts (green) was visualized by immunostaining of human melanoma sections (indicated by white arrows). Sections were co‐stained with a fibroblast‐specific anti‐TE7 antibody. A–B. H&E‐stained melanoma tissue sections. (C) *β*‐catenin immunostaining for infiltrated fibroblasts inside melanoma tumor stroma. (D) *β*‐catenin immunostaining for stromal fibroblasts surrounding invasive melanoma tumor. Image shown is typical of at least 10 human melanoma samples. Scale bar: 100 *μ*m. (E) Percentages of nuclear expression and cytoplasmic expression of *β*‐catenin in TE7‐positive melanoma‐associated fibroblasts are showed as the mean ± SEM, *n* = 3.

*β*‐catenin is involved in many different cellular processes, such as cell growth and differentiation. It has been reported that stromal fibroblasts located in and around breast carcinomas frequently expressed nuclear *β*–catenin 10. To assess *β*‐catenin activity in melanoma‐associated fibroblasts, we co‐stained human melanoma tissue paraffin sections with an anti‐*β*‐catenin antibody and an anti‐fibroblast antibody TE7 18. Interestingly, stromal fibroblasts located in (Fig. 1C) and around (Fig. 1D) invasive melanoma lesion expressed high levels of cytoplasmic and nuclear *β*‐catenin. As shown in Figure 1E, 67.9 ± 9.10% TE7‐positive melanoma‐associated fibroblasts expressed *β*‐catenin while 32.47 ± 3.18% of them were nuclear localized, suggesting stromal *β*‐catenin activity is involved during melanoma formation and development.

### Ablation of *β*‐catenin in dermal fibroblasts causes cell cycle arrest, suppresses cell growth, and reduces the production of chemical factors and ECM proteins

**Figure 2:**
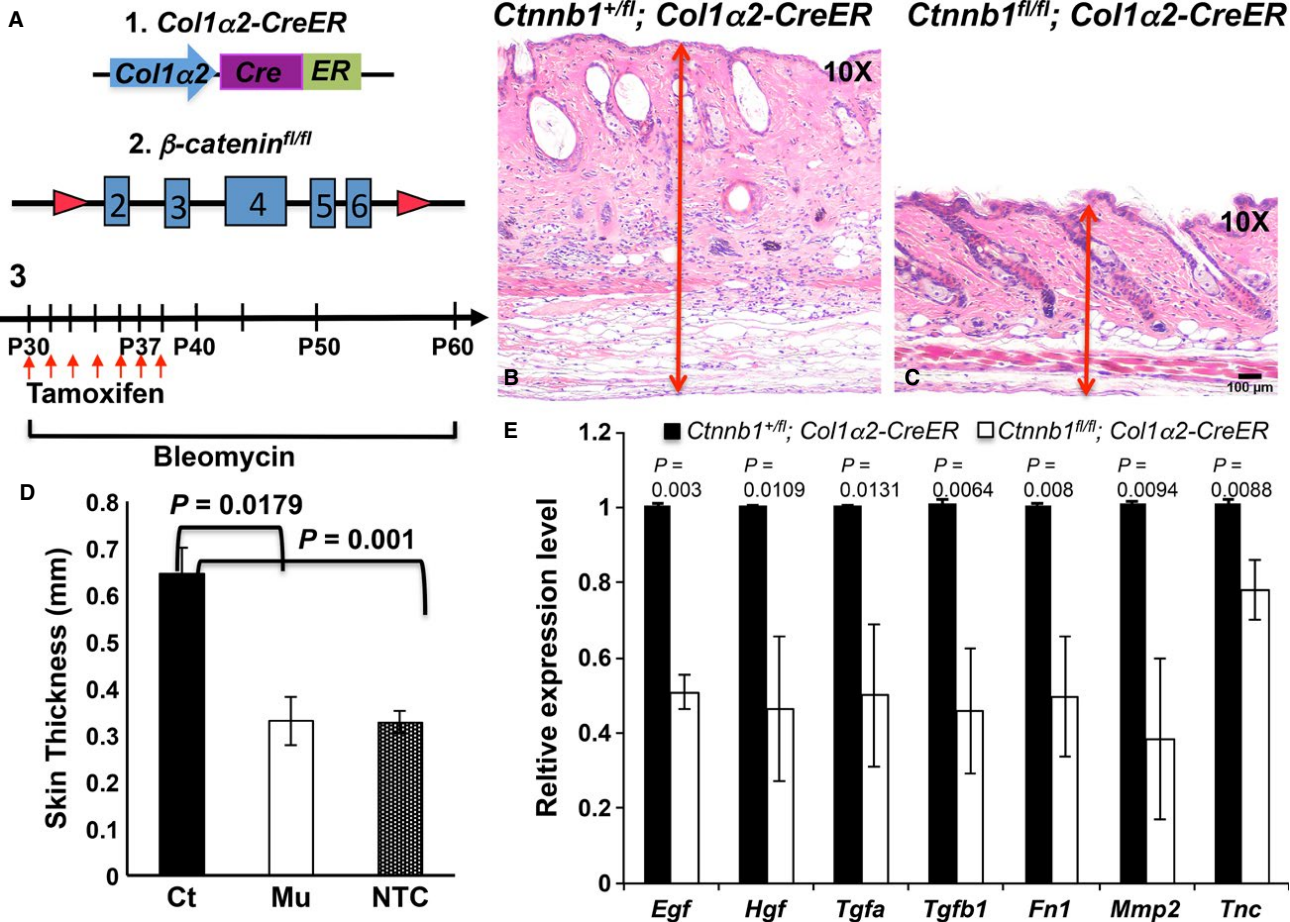
*In vivo β*‐catenin ablation in dermal fibroblasts inhibits abnormal activation in the bleomycin‐induced skin fibrosis. (A) Overview of transgenic constructs and scheme of induction. (A1) The illustration of the *Col1α2‐CreER* transgene construct, which drives the Cre recombinase expression in Col1*α*2‐active fibroblasts. (A2) The illustration of *β*‐catenin genomic locus in *Ctnnb1* ^*fl/fl*^ mouse, which is flanked by 2 loxP sites. (A3) The flow chart is the time scheme for tamoxifen and bleomycin administration. Briefly, *Ctnnb1* ^*fl/fl*^ *, Col1a2‐CreER* mice and control littermates received daily subcutaneous injections of bleomycin for 30 days and i.p. injection of tamoxifen for 7 days starting from P30. Dorsal skin biopsies were harvested for examination at P60. (B‐C) H&E‐stained skin sections from control *Ctnnb1* ^*+/fl*^ *; Col1α2‐CreER* mouse and mutant *Ctnnb1* ^*fl/fl*^ *; Col1α2‐CreER* mouse. (D) Quantification of average dermal thickness of *Ctnnb1* ^*+/fl*^ *; Col1α2‐CreER* mice (Ct), mutant *Ctnnb1* ^*fl/fl*^ *; Col1α2‐CreER* mice (Mu) and untreated wild‐type mice (NTC). Results represent mean ± SEM., *n* = 3, 30 sites/per mouse. *P < 0.05. (E) Gene expression analysis of chemical factors and extracellular matrix (ECM) proteins produced by dermal fibroblasts using qPCR. Total RNA was extracted from dermis of control *Ctnnb1* ^*+/fl*^ *; Col1α2‐CreER* and mutant *Ctnnb1* ^*fl/fl*^ *; Col1α2‐CreER* mouse. Data are representative of three independent experiments and calculated as mean ± SEM *P < 0.05. Scale bar: 100 *μ*m

To determine if *β*‐catenin loss could potentially deactivate stromal fibroblasts and ablate their biological activities, we have introduced a conditional gene knockout mouse model, *Col1α2‐CreER* mouse (Fig. 2A–1), which drives the specific expression of a fusion protein (CreER) combining the Cre recombinase and a mutated ligand‐binding domain of the human estrogen receptor under the control of a fibroblast‐specific *Col1α2* promoter 19. The *Col1α2* gene normally functions in metabolically active fibroblasts during wound healing, fibrosis, and tumor formation. Cre recombinase expressed from the *CreER* gene can only be activated to induce site‐specific recombination upon treatment with tamoxifen or 4‐OHT 20. The combination of the *Col1α2‐CreER* transgene with *β*‐catenin loss‐of‐function alleles allows targeted ablation of *β*‐catenin expression in stromal fibroblasts when induced either *in vivo* or *in vitro*.

**Figure 3:**
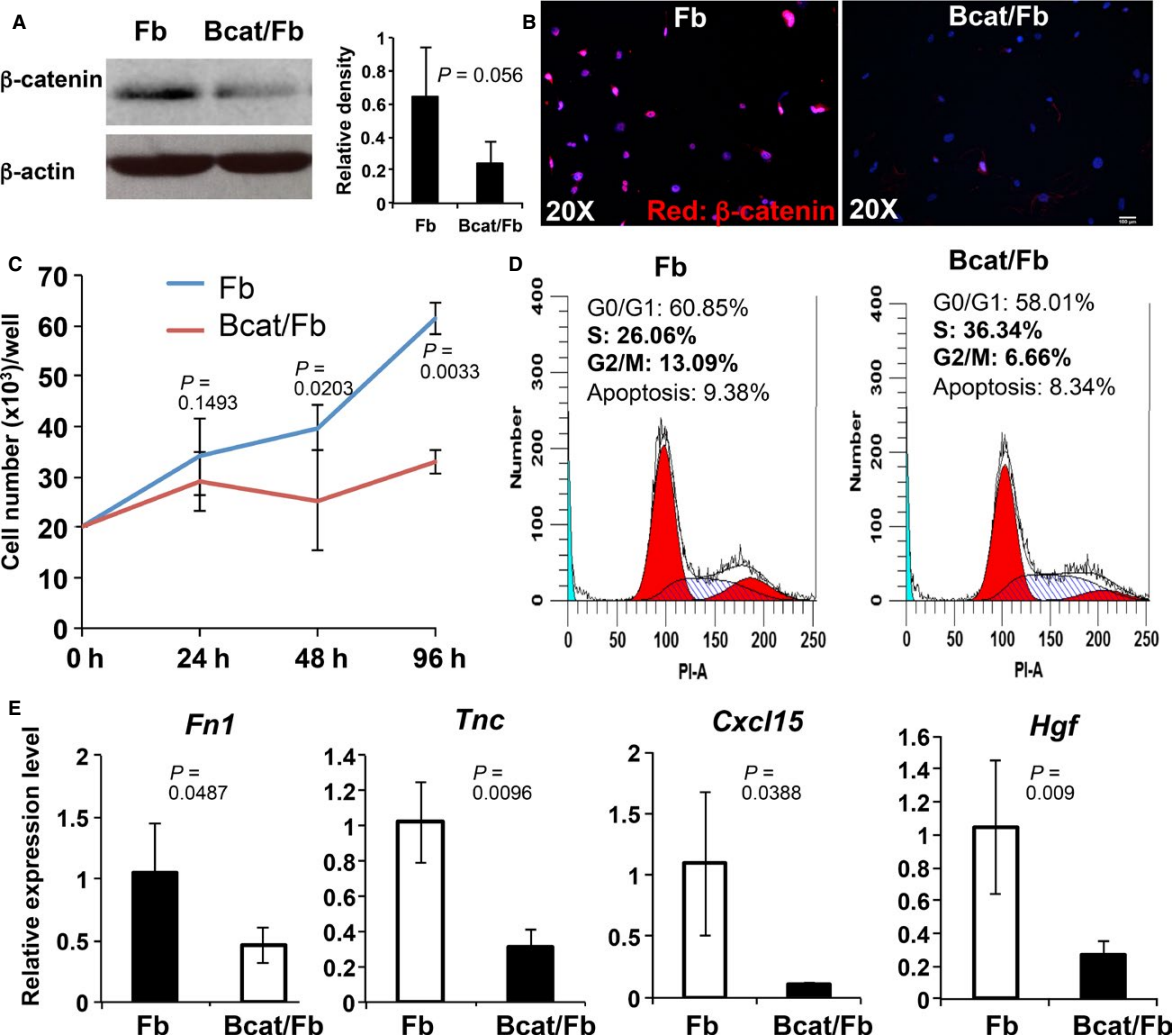
Loss of *β*‐catenin in dermal fibroblasts suppresses cell growth and reduces the production of chemical factors and ECM proteins. Dermal fibroblasts were separated from the dermis of 4‐day‐old *Ctnnb1* ^*fl/fl*^ *, Col1a2‐CreER* (Bcat/Fb), and control *Ctnnb1* ^*+/fl*^ *; Col1α2‐CreER* (Fb) mice and cultured in Dulbecco's Modified Eagle's Medium (DMEM) medium for 2 passages. Purified fibroblasts were then seeded at a density of 3 × 10^5^ cells/dish in 10 cm tissue culture dish and treated with 100 ng/mL 4‐OHT for 48 h. (A) Left: Greatly decreased *β*‐catenin expression in mutant *Ctnnb1* ^*fl/fl*^ *, Col1a2‐CreER* fibroblasts (Bcat/Fb) was confirmed by western blot analysis. Right: The intensity of *β*‐catenin band was shown by scanning X‐ray film and normalized to *β*‐actin band. (B) immunostaining with an anti‐ *β*‐catenin antibody showed the loss of *β*‐catenin expression in a majority of Bcat/Fb fibroblasts. (C) Growth of Bcat/Fb and control Fb fibroblasts was compared by cell number counting. Briefly, 20,000 control or mutant cells were seeded in one well of 24‐well plate (3 repeats) and grew for a period of 4 days. Cells were collected at 24, 48, and 96 h to count numbers using hemocytometer. Data are representative of three independent experiments. (D) Cell cycle analysis of Fb and Bcat/Fb fibroblasts after 48‐h culture by flow cytometry indicating percentages of cells at different phases of cell cycle based on DNA content. *n* = 3. (E) Gene expression analysis of chemical factor and extracellular matrix (ECM) protein genes produced by Fb and Bcat/Fb dermal fibroblasts. Data are representative of at least three independent experiments. Fibroblasts isolated from individual P40 mice were analyzed for gene expression by qPCR (mean ± SEM, *n* = 5). The *Y*‐axis represents fold change in expression with wild‐type levels set to 1.

We generated a double transgenic mouse model, *Ctnnb1* ^*fl/fl*^ *; Col1α2‐CreER*, in which floxed *β*‐catenin alleles (Fig. 2A–2) could be specifically recombined to result in a null mutation in stromal fibroblasts upon Cre recombinase activation. Considering dermal fibroblasts are normally quiescent, we used bleomycin to treat mice and induce fibrosis. This is a widely used approach to mimic human scleroderma in mouse, a disease closely linked to abnormal activation of fibroblasts 21. As depicted in Figure 2A–3, we i.p. injected experimental mice with tamoxifen to induce the expression of Cre recombinase at P30 for 7 days, which subsequently led to the *in vivo β*‐catenin ablation in active *Ctnnb1* ^*fl/fl*^ *; Col1α2‐CreER* fibroblasts. Bleomycin was simultaneously administered to all mice by subcutaneous injection for 30 days. At P60, skins of *Ctnnb1* ^*fl/fl*^ *; Col1α2‐CreER* mutant mice and control littermates were collected for various analyses. As shown in Figure 2C, the dermis of *Ctnnb1* ^*fl/fl*^ *; Col1α2‐CreER* mutant mouse was more than 50% thinner than that of control *Ctnnb1* ^*+/fl*^ *; Col1α2‐CreER* mouse (Fig. 2B). Measurement of skin thickness revealed a mean of 0.65 mm ± 0.05 mm thickness in control mice (*n* ≥ 3), compared with 0.35 mm ± 0.05 mm in *Ctnnb1* ^*fl/fl*^ *; Col1α2‐CreER* mutant mice (*n* ≥ 3) (Fig. 2D). Interestingly, average skin thickness of bleomycin‐treated *Ctnnb1* ^*fl/fl*^ *; Col1α2‐CreER* mutant mice was comparable to that of nontreated wild‐type mice (Fig. 2D), suggesting *β*‐catenin ablation in dermal fibroblasts at least partially rescued the scleroderma phenotype induced by bleomycin. Furthermore, there was less collagen production in mutant dermis than that in control dermis (data now shown). Collagen protein is mainly produced by skin fibroblasts. qPCR showed a significant decrease in production of representative cytokines, growth factors, and ECM proteins in dermis of mutant mice when compared with control mice (Fig. 2E), including epidermal growth factor (EGF), hepatocyte growth factor (HGF/SF), transforming growth factor alpha (TGF‐*α*), transforming growth factor beta1 (TGF‐*β*1), matrix metalloproteinase 2 (MMP2), tenascin C (TN‐C), and fibronectin 1 (FN1). TN‐C and FN1 are pro‐survival ECM proteins normally secreted by stromal fibroblasts 13, 22. MMP2 is known to be produced by normal fibroblasts to regulate and restrain the change of the ECM in healthy tissue 23. Our results suggested that genetic ablation of *β*‐catenin in fibroblasts *in vivo* indeed prevented dermal fibroblasts from being activated by bleomycin stimulation and abrogated their biological functions.

To establish primary murine fibroblasts for *in vitro* and *in vivo* tumor microenvironment investigation, dermal fibroblasts were isolated from neonatal mice carrying either *Ctnnb1* ^*fl/fl*^ *; Col1α2‐CreER* (bcat/Fb) or *Ctnnb1* ^*+/fl*^ *; Col1α2‐CreER* (Fb) alleles for culture as described 24. After 2 passages, the expression of platelet derived growth factor receptor‐alpha (PDGFR‐*α*), which is a surface marker for dermal fibroblasts 25, was examined by flow cytometry to determine the purity of fibroblasts. The result showed that over 98.7% of cultured cells were PDGFR‐*α*‐positive fibroblasts. Fibroblasts were then seeded at a density of 3 × 10^5^ cells/dish in 10 cm tissue culture dish. Ablation of *β*‐catenin in *Ctnnb1* ^*fl/fl*^ *; Col1α2‐CreER* fibroblasts was induced by adding 4‐OHT to the culture medium at a concentration of 100 ng/mL. Control *Ctnnb1* ^*fl/+*^ *; Col1α2‐CreER* fibroblasts underwent the same treatment. After 48‐h incubation, 4‐OHT was removed and replaced with fresh medium with 10% FBS. Ablation of *β*‐catenin expression in mutant fibroblasts was confirmed by western blotting (left in Fig. 3A). *β*‐catenin expression shown as the intensity of *β*‐catenin band normalized to *β*‐actin expression indicated almost a 3‐time reduction in *β*‐catenin‐deficient *Ctnnb1* ^*fl/fl*^ *; Col1α2‐CreER* fibroblasts after 2‐day 4‐OHT induction (right in Fig. 3A). Nuclear translocation of *β*‐catenin protein could no longer be detected in mutant fibroblasts as observed by immunofluorescence staining using an anti‐*β*‐catenin antibody (Fig. 3B). After induction with 4‐OHT, cells were reseeded at a density of 2 × 10^5^/dish in 6 cm culture dish for 3 days in normal medium. Both *Ctnnb1* ^*fl/fl*^ *; Col1α2‐CreER* mutant and *Ctnnb1* ^*+/fl*^ *; Col1α2‐CreER* control fibroblasts were collected every 24 h for cell number counting and cell cycle analysis. *Ctnnb1* ^*fl/fl*^ *; Col1α2‐CreER* mutant fibroblasts growth was significantly inhibited as compared with control fibroblasts (Fig. 3C). Cell cycle analysis revealed that *β*‐catenin loss prevented cell cycle progression in *β*‐catenin‐deficient fibroblasts from the S (synthesis phase) phase to G2 (second gap phase) of the cell division cycle (Fig. 3D). Moreover, qPCR results showed similar reduction in growth factor and ECM protein expression in mutant fibroblasts as data collected from the skin of mutant mice of *in vivo* skin fibrosis model (Fig. 3E). All together, these *in vivo* and *in vitro* data demonstrated that *β*‐catenin is an ideal and effective target to deactivate stromal fibroblasts.

### *In vivo* melanoma formation is significantly accelerated when dermal fibroblasts are deactivated

Based on the above data, we decided to use targeted ablation of *β*‐catenin as an approach to deactivate dermal fibroblasts in order to study their roles during different stages of melanoma development. The *Ctnnb1* ^*fl/fl*^ *; Col1α2‐CreER and Ctnnb1* ^*+/fl*^ *; Col1α2‐CreER* mice were backcrossed to the B6 genetic background. Therefore, cell mixtures of B16F10 murine melanoma cells with either bcat/Fb fibroblasts (*β*‐catenin‐deficient group: B16F10; bcat/Fb) or Fb fibroblasts (control group: B16F10; Fb) can be injected intradermally into the flanks of B6 mice to induce melanoma tumor formation without immune rejection. The B16F10 mouse melanoma line is widely used as a model system for cancer biology research because of its highly invasive and metastatic potential 26.

**Figure 4:**
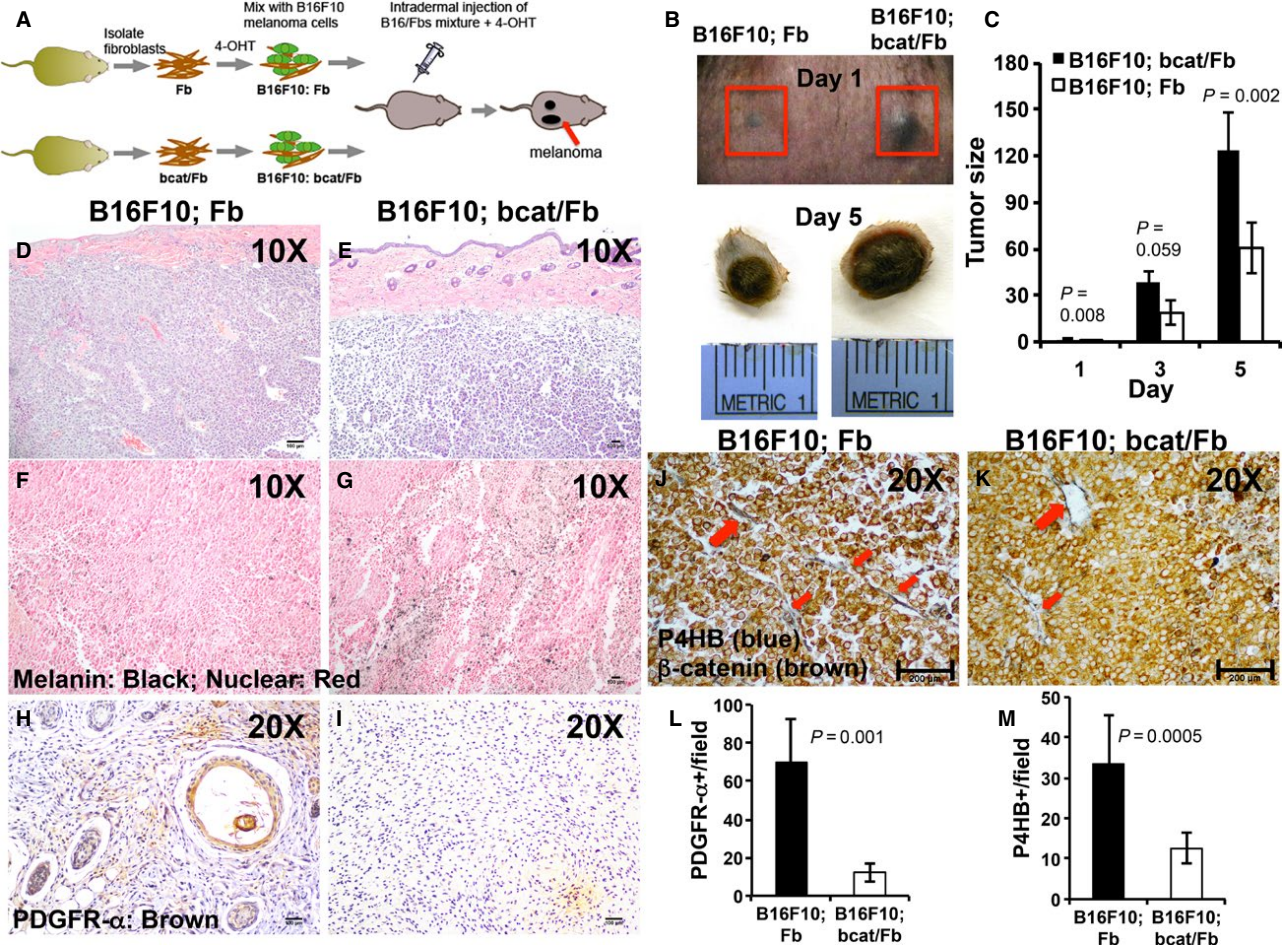
Deactivated dermal fibroblasts leads to accelerated early stage B6F10 melanoma development *in vivo*. (A) Schematic diagram of genetic model for studying *in vivo* effects of deactivation of stromal fibroblasts on melanoma initiation. B16F10 melanoma cells (green) mixed with pretreated control Fb or mutant Bcat/Fb fibroblasts (brown) were injected intradermally into the left and right flanks of same B6 mice to induce melanoma formation for comparison. (B) Reprehensive pictures of melanoma tumors formed from B16F10; Fb and B16F10; bcat/Fb mixtures on Day 1 (B) and Day 5 (C). The day when control tumor was visible on the mice was considered as Day 1. Day 5 was picked as the end point for the study of onset stage of melanoma formation. (C) Tumor size was measured every other day and compared between control B16F10; Fb group and mutant B16F10; bcat/Fb group (*n* = 10). (D–E) H&E‐stained melanoma tumor sections were photographed under light microscope (D: Ct; E: Mu). (F–G) Melanin staining in melanoma tumor sections that were counterstained with nuclear red (F: Ct; G: Mu). H–I. Deactivation of fibroblasts in tumor samples were confirmed by immunostaining with fibroblasts marker PDGFR‐α (H: Ct; I: Mu). (J–K) Melanoma tumor sections were double stained for the expression of an activated fibroblast marker P4HB and *β*‐catenin (K: Ct; L: Mu). Scale bar: (D–I)100 *μ*m; J–K, 200 *μ*m. (L) Number of PDGFR‐*α* positive cells per field (20×) is showed as the mean ± SEM, *n* = 3. (M) Number of P4HB positive cells per field (20×) is showed as the mean ± SEM, *n* = 3.

To determine the potential effects of deactivated stromal fibroblasts on melanoma onset, the B16F10 mice melanoma cells were mixed with 4‐OHT pretreated Fb (B16F10; Fb) or bcat/Fb fibroblasts (B16F10; bcat/Fb) at the ratio of 1:2 and injected intradermally into left or right flank of wild‐type B6 mice to induce melanoma formation as illustrated in Figure 4A. Tamoxifen was i.p. administered to experimental mice for 7 consecutive days after injection. The size of tumors on each mouse was measured and recorded every other day starting from the day when the tumor appeared. Tumor growth rate was calculated, and the difference between the *β*‐catenin‐deficient group and the control group was compared using student's *t*‐test. The mice were sacrificed 2 weeks after tumor grafting, and tumors were collected for analysis. All tumors were encapsulated. We did not detect any local invasion or remote metastasis because the period of tumor development was limited – within a 2‐week time frame.

In general, it took 5 days for B16F10; bcat/Fb mixture to form a melanoma tumor while mixture of B16F10; Fb needed an average of 7–8 days. For the convenience of tumor size comparison and calculation, we consider the day when the B16F10; Fb tumor appeared as day 1. As shown in Figure 4B, the B16F10; bcat/Fb tumor appeared bigger than the B16F10; Fb tumor at day 1. At the end time point (day 5 in Fig. 4B), the B16F10; bcat/Fb tumor remained bigger and heavier than the B16F10; Fb tumor. It has been consistently observed that formation of melanoma tumors was always significantly accelerated with the B16F10; bcat/Fb mixture (Fig. 4C). Histological analysis showed that there were no clear structural and histological differences between B16F10; bcat/Fb (Fig. 4E) and B16F10; Fb tumors (Fig. 4D). Interestingly, it appeared that B16F10; bcat/Fb tumors had more melanin contents (Fig. 4G) than B16F10; Fb tumors (Fig. 4F). Analysis of expression of PDGFR‐*α* 27 and prolyl 4‐hydroxylase beta polypeptide (P4HB) 28, which are markers for fibroblasts and activated fibroblasts respectively, showed that there were fewer fibroblasts (Fig. 4I and L) and activated fibroblasts (Fig. 4K and M) in B16F10; bcat/Fb tumors than those in B16F10; Fb tumors. Taken together, our results showed that NFs block the onset of melanoma tumor unless they are deactivated.

### Dermal fibroblasts induce a G1/S cell cycle arrest in melanoma cells through the MAPK/ERK and Rb signaling pathways at the onset of melanoma formation

**Figure 5:**
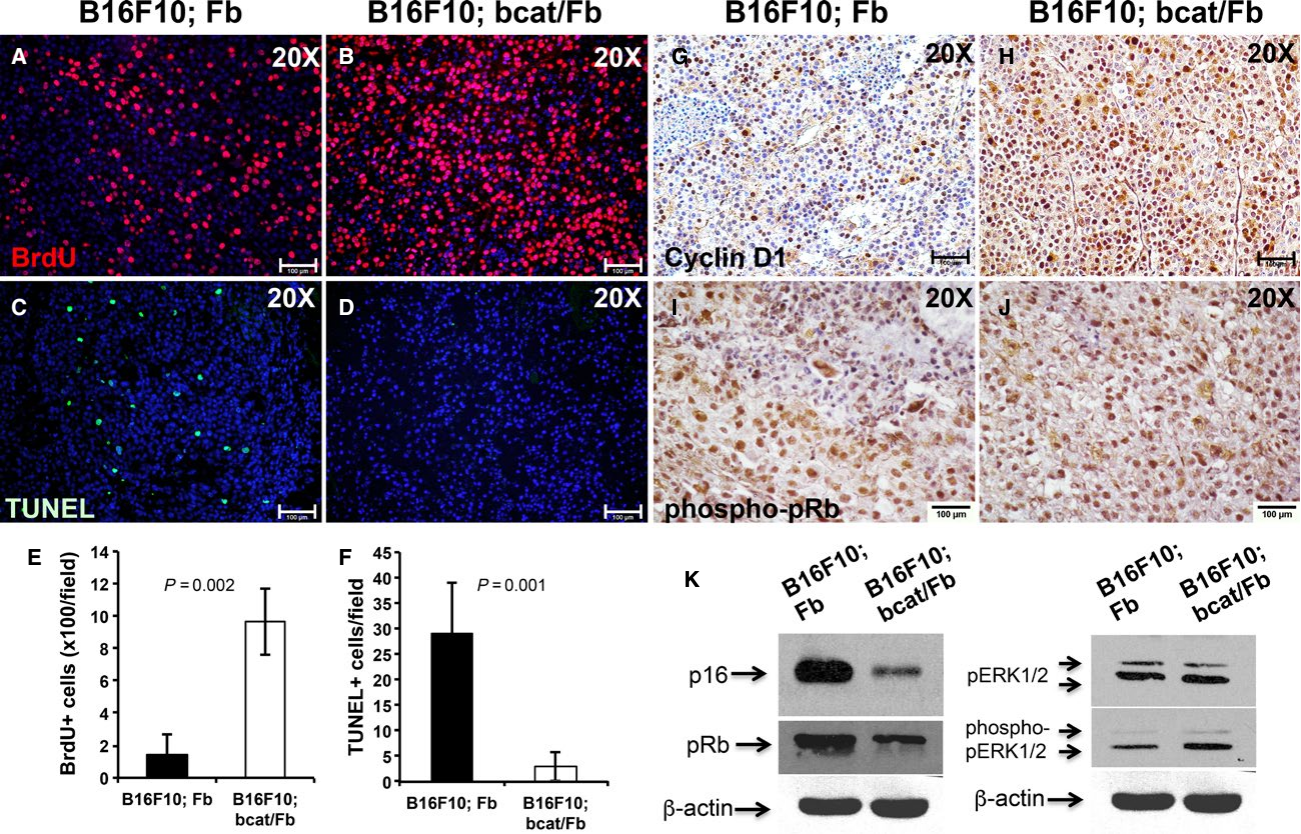
Dermal fibroblasts suppress early stage melanoma tumor growth by inducing a cell cycle arrest in tumor cells. Cell proliferation in control (A) and mutant tumors (B) was examined by BrdU labeling and immunostaining. To detect cell apoptosis in control (C) and mutant (D) tumors, TUNEL staining was performed. (E) Number of BrdU positive cells per field (20×) is showed as the mean ± SEM, *n* = 3. (F) Number of TUNEL positive cells per field (20×) is showed as the mean ± SEM, *n* = 3. Reduced immunostaining of Cyclin D1 was detected in B16F10; Fb tumor (G) as compared with B16F10; bcat/Fb tumor sample (H). phospho‐pRb expression was evaluated on paraffin sections of B16F10; Fb tumor (I) and B16; bcat/Fb tumor (J) by immunohistochemistry. Images shown are representative of 5 pairs of melanoma samples. (K) Expression of p16, pRb, pERK1/2, and phospho‐pERK1/2 protein was analyzed and compared between B16F10; Fb and B16; bcat/Fb tumors by western blotting. *β*‐actin was used as internal control. Data are representative of three independent experiments. Scale bar: A–D and G–J,100 *μ*m.

To determine the underlying cause of the suppressed melanoma formation by normal dermal fibroblasts, we assayed for changes in cell proliferation and apoptosis. As shown in Figure 5E, immunofluorescence staining for Bromodeoxyuridine (BrdU) labeling as a cell proliferation marker revealed greatly increased cell proliferation in B16F10; bcat/Fb melanoma tumors (Fig. 5B) compared with control tumors (Fig. 5A). Terminal deoxynucleotidyl transferase dUTP nick end labeling (TUNEL) assay showed that there were less apoptotic cells in B16F10; bcat/Fb tumors (Fig. 5D and F) than in control ones (Fig. 5C). We also examined cell senescence state in melanoma tumors by analyzing SA‐*β*‐Gal staining. In line with the slow melanoma formation from B16F10; Fb cell mixture, B16F10; Fb tumors had more senescent cells than B16F10; bcat/Fb tumors (data now shown).

Cyclin D1 directly regulates cellular proliferation by initiating the transition from late G1 to S phase of the cell cycle 29. Immunostaining results showed significant upregulated expression of cyclin D1 in B16F10; bcat/Fb tumors (Fig. 5H) as compared with B16F10; Fb tumors (Fig. 5G), suggesting that there was an accelerated G1 to S phase cell cycle progression in B16F10; bcat/Fb melanoma tumors when stromal fibroblasts were deactivated through *β*‐catenin ablation. Cyclin D1 forms a complex with cyclin‐dependent kinase CDK4/6 resulting in the sequestration of the CDK inhibitors, p16(INK4a), p21(cip1), and p27(kip1) 30. p16 also contributes to irreversible cell growth arrest process 31. Western blot analysis showed a dramatic reduction of p16 expression in B16F10; bcat/Fb melanoma tumors (Fig. 5K). Cyclin D1 is a direct target of the mitogen‐activated protein kinase (MAPK)/extracellular signal‐regulated kinase (ERK) signaling. Upregulation of phospho‐pERK1/2 in tumor tissue with *β*‐catenin‐deficient fibroblasts was confirmed by western blots (Fig. 5K). The pocket proteins, comprising the retinoblastoma protein Rb (pRb), p107, and p130, function to prevent excessive cell growth by inhibiting cell cycle progression. Our results showed pRb expression in B16F10; bcat/Fb melanoma tumors was downregulated (Fig. 5K) while the expression of phopho‐pRb was increased (Fig. 5I and J), possibly contributing to the abnormal cell cycle progression in melanoma cells. Taken together, these results suggested that normal dermal fibroblasts induced a G1/S cell cycle arrest in melanoma cells during the early tumor development stage through the MAPK/ERK and Rb signaling pathways while deactivation of dermal fibroblasts by *β*‐catenin ablation could potentially abrogate their suppressive functions.

### Dermal fibroblasts prevent epithelial–mesenchymal transition in melanoma cells at the melanoma onset

**Figure 6:**
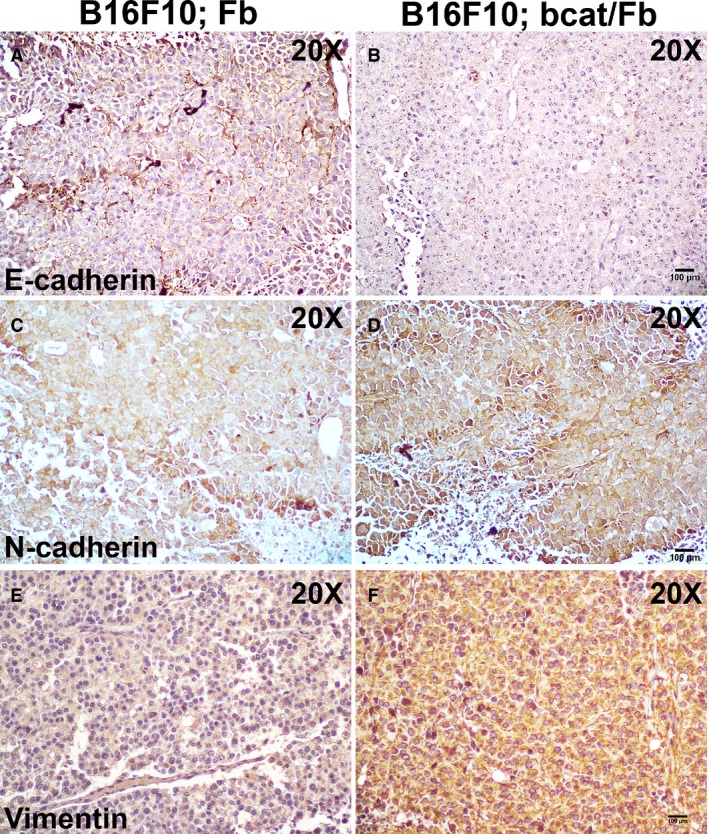
Dermal fibroblasts suppress epithelial‐mesenchymal transition during the onset of melanoma formation. Expression of E‐cadherin, N‐cadherin, and Vimentin were evaluated on paraffin sections of B16F10; Fb tumor (A, C, E) and B16; bcat/Fb tumor (B, D, F) by immunohistochemistry. Images shown are representative of three pairs of melanoma samples. Scale bar:100 *μ*m

Epithelial–mesenchymal transition (EMT) occurs in most tumorigenic cells including melanoma cells, resulting in increased tumor invasion and metastasis 32. It has been suggested that mesenchymal markers such as vimentin and N‐cadherin are upregulated while epithelial markers such as E‐cadherin are downregulated when cancer cells undergo the EMT process 33. We discovered that tumor cells had decreased E‐cadherin expression (Fig. 6B) and increased N‐cadherin and Vimentin expression (Fig. 6D and F) in melanoma tumors formed from B16F10; bcat/Fb mixture. This finding provided another possible cause that deactivation of normal dermal fibroblasts by *β*‐catenin ablation allows significantly rapid melanoma development *in vivo*. On the other hand, the results indicated that dermal fibroblasts actually crosstalk with melanoma cells to modulate EMT at the initial stage of melanoma formation.

## Discussion

Melanomas are well known for their aggressive clinical behavior, high propensity to metastasize, and resistance to therapeutic treatments. Clinical studies have demonstrated that melanomas arise from pigment‐producing melanocytes due to genetic mutations. Normal melanocytes reside predominantly at the junction of the epidermis and the dermis forming an “Epidermal Melanin Unit” with up to thirty‐six keratinocytes. It has been reported that melanocytes are restricted in the epidermis due to the E‐cadherin‐mediated interactions with keratinocytes 34. During the process of melanoma development and metastasis, malignant melanoma cells need to penetrate the basement membrane and invade the underlying dermis in order to spread to other parts of the body 35. The switch from E‐cadherin to N‐cadherin expression controlled by PTEN/PI3K signaling in melanoma cells allows them to break away from keratinocytes and bind to N‐cadherin‐expressing fibroblasts 36, 37, 38. Clearly, malignant melanoma cells interact actively and extensively with dermal fibroblasts, which undoubtedly could serve as a major source for CAFs and play an important role in melanoma development and metastasis.

Dermal fibroblasts generally appear to be quiescent under normal healthy conditions and function as required to maintain tissue homeostasis 39. They exhibit both phenotypical, molecular, and biochemical differences from CAFs 6. When the tissue experiences tumorigenesis, resident fibroblasts are not in a state to support tumor cell proliferation and expansion. It is widely believed that they need to be recruited and stimulated by melanoma cells to enter a continuous state of irreversible activation and transdifferentiate into CAFs 40. Following their abnormal transformation, CAFs will produce chemical factors and remodel the tumor microenvironment to establish an environment conducive for melanoma growth and metastasis. However, it remains unclear as to how normal dermal fibroblasts interact with melanoma cells to support or suppress the growth and progression of early stage malignant lesions.

In order to determine the role and function of normal dermal fibroblasts at the initiation stage of melanomagenesis, we have designed a novel genetic approach to deactivate dermal fibroblasts that could be manipulated at different stages of melanoma formation. The biological activities of dermal fibroblasts can be regulated by an array of cellular signaling pathways, including Wnt/*β*‐catenin signaling, TGF signaling, Notch signaling, etc. Wnt/*β*‐catenin signaling is one of the key signaling pathways that are involved in a variety of cellular processes such as proliferation, apoptosis, migration, and inflammation. It has been shown that Wnt/*β*‐catenin signaling plays an important role in fibroblast growth 41 and differentiation of stromal fibroblasts into activated or myofibroblasts in wound healing 42 and breast cancer development 10. Several groups have previously confirmed that the upregulation of *β*‐catenin activity in mouse dermal fibroblasts resulted in abnormally activated phenotype that is a resemblance to human fibrosis 11, 43. Here, we show that nuclear *β*‐catenin expression was detected in stromal fibroblasts surrounding and infiltrating human melanoma stroma, suggesting *β*‐catenin signaling possibly plays an important role in stromal fibroblasts in the melanoma tumor microenvironment.

Transgenic *Col1α2‐CreER* mouse model drives the unique expression of Cre recombinase in dermal fibroblasts under the control of the fibroblast‐specific *Col1α2* promoter, which is most active in dividing fibroblasts such as during wound healing, fibrosis, and tumor formation 19. Cre recombinase functions *in vivo* to remove a specific DNA fragment that is flanked by loxP sites but is not active unless mouse is treated with tamoxifen or 4‐OHT. The combination of this *Col1α2‐CreER* mouse with *β*‐catenin loss‐of‐function *Ctnnb1* ^*fl/fl*^ mouse enables the inducible ablation of *β*‐catenin activity in dermal fibroblasts *in vitro* or *in vivo*.

By studying this mouse model in bleomycin‐induced skin fibrosis, we discovered that the dermis of *β*‐catenin‐deficient mice appeared much thinner than that of control littermates after bleomycin stimulation, suggesting proliferation of dermal fibroblasts was inhibited. Interestingly, the production of collagen fiber, chemical factors, and ECM proteins that are normally generated by dermal fibroblasts were also greatly affected. *In vitro* culture of *β*‐catenin‐deficient fibroblasts and normal fibroblasts showed same phenotypes as *in vivo* observation. *β*‐catenin‐deficient fibroblasts grew much slower than normal fibroblasts. Further cell cycle analysis indicates that there was a cell cycle arrest in dermal fibroblasts upon *β* ‐catenin ablation. Our results suggest that *β*‐catenin depletion is a useful tool to deactivate dermal fibroblasts *in vivo*. Targeted ablation of stromal *β*‐catenin could be applied to study the cellular role and function of stromal fibroblasts at different stages of melanoma formation.

By grafting mixture of B16F10 melanoma cells and dermal fibroblasts, we have a valid approach to determine how resident fibroblasts function at the initial stage of melanoma formation. Our work showed that disruption of *β*‐catenin in dermal fibroblasts associated with melanoma cells indeed deactivated them, and this deactivation actually allowed B16F10 melanoma tumor to grow faster as compared with melanoma tumor formed with normal fibroblasts. This result is possibly contrary to existing concepts but not completely unexpected. Although it is widely accepted that stromal fibroblasts are involved in promoting tumor initiation, growth and metastasis in many different types of solid tumors, including melanoma, some papers suggested that stromal fibroblasts initially were not cancer‐supportive and exhibited suppressive effects, and only promoted tumor progression when abnormally activated to become CAFs 44. Consistent with *in vivo* and *in vitro β*‐catenin ablation in fibroblasts experiments, few fibroblasts could be detected in B16F10; bcat/Fb tumor samples. This reduction consequently diminished the inhibitory effects of dermal fibroblasts on melanoma tumor formation. Our findings provide clear evidences that dermal fibroblasts fight against melanoma tumor growth at the onset stage.

Melanoma cells and dermal fibroblasts communicate throughout the tumorigenic process. Although genetic mutations in melanoma cells cause regulatory mechanisms that monitor DNA damage, repair and cell cycle progression to malfunction, our experimental results also revealed that the tumor microenvironment including CAFs could potentially send positive or negative signals to tumor cells to influence their biological behaviors. When dermal fibroblasts were deactivated by *β*‐catenin ablation and no longer able to release enough inhibitory signals, melanoma showed increased proliferation, decreased cell senescence, and apoptosis. Uncontrolled cell cycle is a major driver of unrestricted tumor cell growth. In our tumor grafting experiment, B16F10; bcat/Fb melanoma tumors showed upregulated expression of cyclin D1 and decreased expression of CDK inhibitor p16 as compared with B16F10; Fb melanoma. Cyclin D1 is a direct target of the MAPK/ERK signaling pathways, which controls cellular processes such as cell proliferation and migration in cancer development 45. Upregulation of phospho‐pERK1/2 expression in B16F10; bcat/Fb melanoma tumors was confirmed by western blots. Furthermore, the expression of pRb protein was greatly reduced in B16F10; bcat/Fb melanoma tumors while phospho‐pRb expression was increased, suggesting the pRb signaling pathway and associated inhibitory effects on cell cycle progression played an important role in the suppression of melanoma formation by dermal fibroblasts. Those findings clearly showed crosstalk between melanoma cells and stromal fibroblasts could regulate the cellular signaling pathways in tumor cells and modulate the phenotype of melanoma tumors.

One hallmark of cancer is that tumor cells progressively acquire the abilities to escape primary tumor sites, invade adjacent tissues and colonize at distant sites 46, 47. Deactivation of stromal fibroblasts at the tumor onset stage causes the destruction of histological structure of dermal tissue, which in turn promotes tumor cells to grow and migrate. Our data also showed that one of the most important cell‐cell adhesive molecules, E‐cadherin, was downregulated in melanoma cells when dermal fibroblasts were deactivated, potentially resulting in increased tumor cell growth and motility. In contrary, the expression of vimentin, a mesenchymal marker of cells undergoing EMT during metastatic progression, was upregulated in melanoma cells. Clinical study on human melanoma samples showed lower E‐cadherin expression and higher vimentin expression, which is associated with overexpression of phospho‐pERK, which enhanced the metastatic and invasive potential of melanoma 48, 49. Patsy et al. suggested in their paper that cell invasiveness was significantly facilitated after losing E‐cadherin expression and expressing mesenchymal markers such as vimentin in MCF‐7 breast cancer cells 50. Our findings provide another explanation that dermal fibroblasts function to block the malignant onset of melanoma by preventing EMT in melanoma cells. However, how dermal fibroblasts communicate with melanoma cells to regulate cadherin expression and EMT remains to be explored.

Stromal fibroblasts may play distinguished roles before and after interaction with melanoma cells. Our findings provided clear evidence that normal dermal fibroblasts interact with melanoma cells and play a defensive role at the onset of melanoma formation, which happens before they are abnormally activated by stimulatory signals from melanoma cells and acquire tumor‐promoting properties. Upon reciprocal crosstalk with tumor cells, their role and functions may change and become supportive of tumor progression and invasion. On the other hand, *β*‐catenin signaling may also exert different roles in the biological activities of normal fibroblasts and CAFs. It would be very interesting to understand in future whether deactivation of CAFs during later stage of melanoma development could effectively inhibit malignant expansion of tumor cells and bypass genomic instability considering *β*‐catenin ablation deprives stromal fibroblasts of their biological functions. *β*‐catenin may serve as a potential target for a combination treatment targeting both the host and melanoma tumor cells.

## Conflict of Interest

The authors declare no conflict of interest.

## References

[1] Brychtova S., M. Bezdekova , J. Hirnak , E. Sedlakova , M. Tichy , and T. Brychta . 2011 Stromal microenvironment alterations in malignant melanoma Pp. 335 *in* Murph P. M. Research on melanoma‐a glimpse into current directions and future trendsed. InTech, Rijeka, Croatia.

[2] Li, H. , X. Fan , and J. Houghton . 2007 Tumor microenvironment: the role of the tumor stroma in cancer. J. Cell. Biochem. 101:805–815. 17226777 10.1002/jcb.21159

[3] Xing, F. , J. Saidou , and K. Watabe . 2010 Cancer associated fibroblasts (CAFs) in tumor microenvironment. Front Biosci. 15:166–179. 10.2741/3613 PMC2905156 20036813

[4] Zhou, L. , K. Yang , T. Andl , R. R. Wickett , and Y. Zhang . 2015 Perspective of targeting cancer‐associated fibroblasts in melanoma. J. Cancer 6:717–726. 26185533 10.7150/jca.10865 PMC4504107

[5] Li, G. , K. Satyamoorthy , F. Meier , C. Berking , T. Bogenrieder , and M. Herlyn . 2003 Function and regulation of melanoma‐stromal fibroblast interactions: when seeds meet soil. Oncogene 22:3162–3171. 12789292 10.1038/sj.onc.1206455

[6] Peng, Q. , L. Zhao , Y. Hou , Y. Sun , L. Wang , H. Luo , et al. 2013 Biological characteristics and genetic heterogeneity between carcinoma‐associated fibroblasts and their paired normal fibroblasts in human breast cancer. PLoS ONE 8:e60321. 23577100 10.1371/journal.pone.0060321 PMC3618271

[7] Villanueva, J. , and M. Herlyn . 2008 Melanoma and the tumor microenvironment. Curr. Oncol. Rep. 10:439–446. 18706274 10.1007/s11912-008-0067-y PMC5662003

[8] Madar, S. , I. Goldstein , and V. Rotter . 2013 ‘Cancer associated fibroblasts’ – more than meets the eye. Trends Mol. Med. 19:447–453. 23769623 10.1016/j.molmed.2013.05.004

[9] Flach, E. H. , V. W. Rebecca , M. Herlyn , K. S. Smalley , and A. R. Anderson . 2011 Fibroblasts contribute to melanoma tumor growth and drug resistance. Mol. Pharm. 8:2039–2049. 22067046 10.1021/mp200421k PMC3235959

[10] Verghese, E. T. , H. Shenoy , V. J. Cookson , C. A. Green , J. Howarth , R. H. Partanen , et al. 2011 Epithelial‐mesenchymal interactions in breast cancer: evidence for a role of nuclear localized beta‐catenin in carcinoma‐associated fibroblasts. Histopathology 59:609–618. 22014042 10.1111/j.1365-2559.2011.03917.x

[11] Beyer, C. , A. Schramm , A. Akhmetshina , C. Dees , T. Kireva , K. Gelse , et al. 2012 Beta‐catenin is a central mediator of pro‐fibrotic Wnt signaling in systemic sclerosis. Ann. Rheum. Dis. 71:761–767. 22328737 10.1136/annrheumdis-2011-200568 PMC3951949

[12] Bainbridge, P. 2013 Wound healing and the role of fibroblasts. J. Wound Care 22(407–8):10–12. 10.12968/jowc.2013.22.8.407 23924840

[13] Bielefeld, K. A. , S. Amini‐Nik , H. Whetstone , R. Poon , A. Youn , J. Wang , et al. 2011 Fibronectin and beta‐catenin act in a regulatory loop in dermal fibroblasts to modulate cutaneous healing. J. Biol. Chem. 286:27687–27697. 21652705 10.1074/jbc.M111.261677 PMC3149359

[14] Takashima, A. . Establishment of fibroblast cultures Current protocols in cell biology 1998;Chapter 2:2.1.‐2.1.12. 10.1002/0471143030.cb0201s00 18228346

[15] Zhang, Y. , P. Tomann , T. Andl , N. M. Gallant , J. Huelsken , B. Jerchow , et al. 2009 Reciprocal requirements for EDA/EDAR/NF‐kappaB and Wnt/beta‐catenin signaling pathways in hair follicle induction. Dev. Cell 17:49–61. 19619491 10.1016/j.devcel.2009.05.011 PMC2859042

[16] Whittaker, P. , R. A. Kloner , D. R. Boughner , and J. G. Pickering . 1994 Quantitative assessment of myocardial collagen with picrosirius red staining and circularly polarized light. Basic Res. Cardiol. 89:397–410. 7535519 10.1007/BF00788278

[17] Debacq‐Chainiaux, F. , J. D. Erusalimsky , J. Campisi , and O. Toussaint . 2009 Protocols to detect senescence‐associated beta‐galactosidase (SA‐betagal) activity, a biomarker of senescent cells in culture and in vivo. Nat. Protoc. 4:1798–1806. 20010931 10.1038/nprot.2009.191

[18] Goodpaster, T. , A. Legesse‐Miller , M. R. Hameed , S. C. Aisner , J. Randolph‐Habecker , and H. A. Coller . 2008 An immunohistochemical method for identifying fibroblasts in formalin‐fixed, paraffin‐embedded tissue. J. Histochem. Cytochem. 56:347–358. 18071065 10.1369/jhc.7A7287.2007 PMC2326106

[19] Zheng, B. , Z. Zhang , C. M. Black , B. de Crombrugghe , and C. P. Denton . 2002 Ligand‐dependent genetic recombination in fibroblasts : a potentially powerful technique for investigating gene function in fibrosis. Am. J. Pathol. 160:1609–1617. 12000713 10.1016/S0002-9440(10)61108-X PMC1850857

[20] Metzger, D. , J. Clifford , H. Chiba , and P. Chambon . 1995 Conditional site‐specific recombination in mammalian‐cells using a ligand‐dependent chimeric cre recombinase. Proc. Natl Acad. Sci. USA 92:6991–6995. 7624356 10.1073/pnas.92.15.6991 PMC41457

[21] LeRoy, E. C. 1974 Increased collagen synthesis by scleroderma skin fibroblasts in vitro: a possible defect in the regulation or activation of the scleroderma fibroblast. J. Clin. Invest. 54:880–889. 4430718 10.1172/JCI107827 PMC301627

[22] Chiquet‐Ehrismann, R. , M. Tannheimer , M. Koch , A. Brunner , J. Spring , D. Martin , et al. 1994 Tenascin‐C expression by fibroblasts is elevated in stressed collagen gels. J. Cell Biol. 127:2093–2101. 7528751 10.1083/jcb.127.6.2093 PMC2120287

[23] Lindner, D. , C. Zietsch , P. M. Becher , K. Schulze , H.‐P. Schultheiss , C. Tschöpe , et al. 2012 Differential expression of matrix metalloproteases in human fibroblasts with different origins. Biochem. Res. Int. 2012:10. 10.1155/2012/875742 PMC3303709 22500233

[24] Seluanov, A. , A. Seluanov , and V. Gorbunova . Establishing primary adult fibroblast cultures from rodents. J. Vis. Exp. 2010(44):e2033. 10.3791/2033 PMC3185624 20972406

[25] Sharon, Y. , L. Alon , S. Glanz , C. Servais , and N. Erez . Isolation of normal and cancer‐associated fibroblasts from fresh tissues by Fluorescence Activated Cell Sorting (FACS). J. Vis. Exp 2013(71):e4425. 23354290 10.3791/4425 PMC3582516

[26] Kim, S. H. , Y. Kim , M. Kim , D. S. Kim , S. C. Lee , S. W. Chi , et al. 2009 Comparative proteomic analysis of mouse melanoma cell line B16, a metastatic descendant B16F10, and B16 overexpressing the metastasis‐associated tyrosine phosphatase PRL‐3. Oncol. Res. 17:601–612. 19806791 10.3727/096504009789745494

[27] Donovan, J. , X. Shiwen , J. Norman , and D. Abraham . 2013 Platelet‐derived growth factor alpha and beta receptors have overlapping functional activities towards fibroblasts. Fibrogenesis Tissue Repair 6:10. 23663505 10.1186/1755-1536-6-10 PMC3667071

[28] Malmstrom, E. , M. Sennstrom , A. Holmberg , H. Frielingsdorf , E. Eklund , L. Malmstrom , et al. 2007 The importance of fibroblasts in remodelling of the human uterine cervix during pregnancy and parturition. Mol. Hum. Reprod. 13:333–341. 17337476 10.1093/molehr/gal117

[29] Fu, M. , C. Wang , Z. Li , T. Sakamaki , and R. G. Pestell . 2004 Minireview: cyclin D1: normal and abnormal functions. Endocrinology 145:5439–5447. 15331580 10.1210/en.2004-0959

[30] Klein, E. A. , and R. K. Assoian . 2008 Transcriptional regulation of the cyclin D1 gene at a glance. J. Cell Sci. 121:3853–3857. 19020303 10.1242/jcs.039131 PMC4545630

[31] Rayess, H. , M. B. Wang , and E. S. Srivatsan . 2012 Cellular senescence and tumor suppressor gene p16. Int. J. Cancer 130:1715–1725. 22025288 10.1002/ijc.27316 PMC3288293

[32] Heerboth, S. , G. Housman , M. Leary , M. Longacre , S. Byler , K. Lapinska , et al. 2015 EMT and tumor metastasis. Clin. Trans. Med. 4:6–19. 10.1186/s40169-015-0048-3 PMC4385028 25852822

[33] Voulgari, A. , and A. Pintzas . 2009 Epithelial‐mesenchymal transition in cancer metastasis: mechanisms, markers and strategies to overcome drug resistance in the clinic. Biochim. Biophys. Acta 1796:75–90. 19306912 10.1016/j.bbcan.2009.03.002

[34] Tang, A. , M. S. Eller , M. Hara , M. Yaar , S. Hirohashi , and B. A. Gilchrest . 1994 E‐Cadherin is the major mediator of human melanocyte adhesion to keratinocytes in‐vitro. J. Invest. Dermatol. 102:36–536. 10.1242/jcs.107.4.983 8056851

[35] Gaggioli, C. , and E. Sahai . 2007 Melanoma invasion‐current knowledge and future directions. Pigm. Cell Res. sponsored by the European Society for Pigment Cell Research and the International Pigment Cell Society 20:161–172. 10.1111/j.1600-0749.2007.00378.x 17516924

[36] Li, G. , K. Satyamoorthy , and M. Herlyn . 2001 N‐cadherin‐mediated intercellular interactions promote survival and migration of melanoma cells. Cancer Res. 61:3819–3825. 11325858

[37] Lade‐Keller, J. , R. Riber‐Hansen , P. Guldberg , H. Schmidt , S. J. Hamilton‐Dutoit , and T. Steiniche . 2013 E‐ to N‐cadherin switch in melanoma is associated with decreased expression of phosphatase and tensin homolog and cancer progression. Br. J. Dermatol. 169:618–628. 23662813 10.1111/bjd.12426

[38] Hao, L. , J. R. Ha , P. Kuzel , E. Garcia , and S. Persad . 2012 Cadherin switch from E‐ to N‐cadherin in melanoma progression is regulated by the PI3K/PTEN pathway through Twist and Snail. Br. J. Dermatol. 166:1184–1197. 22332917 10.1111/j.1365-2133.2012.10824.x

[39] Nolte, S. V. , W. Xu , H. O. Rennekampff , and H. P. Rodemann . 2008 Diversity of fibroblasts–a review on implications for skin tissue engineering. Cells Tissues Organs 187:165–176. 18042973 10.1159/000111805

[40] Augsten, M. 2014 Cancer‐associated fibroblasts as another polarized cell type of the tumor microenvironment. Front Oncol. 4:62–70. 24734219 10.3389/fonc.2014.00062 PMC3973916

[41] Soler, C. , C. Grangeasse , L. G. Baggetto , and O. Damour . 1999 Dermal fibroblast proliferation is improved by beta‐catenin overexpression and inhibited by E‐cadherin expression. FEBS Lett. 442:178–182. 9928997 10.1016/s0014-5793(98)01648-2

[42] Poon, R. , S. A. Nik , J. Ahn , L. Slade , and B. A. Alman . 2009 Beta‐catenin and transforming growth factor beta have distinct roles regulating fibroblast cell motility and the induction of collagen lattice contraction. BMC Cell Biol. 10–20:38. 10.1186/1471-2121-10-38 PMC2691404 19432963

[43] Hamburg, E. J. , and R. P. Atit . 2012 Sustained [beta]‐catenin activity in dermal fibroblasts is sufficient for skin fibrosis. J. Invest. Dermatol. 132(10):2469–2472. 22622416 10.1038/jid.2012.155 PMC3430808

[44] Beacham, D. A. , and E. Cukierman . 2005 Stromagenesis: the changing face of fibroblastic microenvironments during tumor progression. Semin. Cancer Biol. 15:329–341. 15970443 10.1016/j.semcancer.2005.05.003

[45] McCubrey, J. A. , L. S. Steelman , W. H. Chappell , S. L. Abrams , E. W. Wong , F. Chang , et al. 2007 Roles of the Raf/MEK/ERK pathway in cell growth, malignant transformation and drug resistance. Biochim. Biophys. Acta 1773:1263–1284. 17126425 10.1016/j.bbamcr.2006.10.001 PMC2696318

[46] Hanahan, D. , and R. A. Weinberg . 2011 Hallmarks of cancer: the next generation. Cell 144:646–674. 21376230 10.1016/j.cell.2011.02.013

[47] Hanahan, D. , and R. A. Weinberg . 2000 The hallmarks of cancer. Cell 100:57–70. 10647931 10.1016/s0092-8674(00)81683-9

[48] Hendrix, M. J. , E. A. Seftor , Y. W. Chu , R. E. Seftor , R. B. Nagle , K. M. McDaniel , et al. 1992 Coexpression of vimentin and keratins by human melanoma tumor cells: correlation with invasive and metastatic potential. J. Natl Cancer Inst. 84:165–174. 1371813 10.1093/jnci/84.3.165

[49] Caramel, J. , E. Papadogeorgakis , L. Hill , G. J. Browne , G. Richard , A. Wierinckx , et al. 2013 A switch in the expression of embryonic EMT‐inducers drives the development of malignant melanoma. Cancer Cell 24:466–480. 24075834 10.1016/j.ccr.2013.08.018

[50] Soon, P. S. , E. Kim , C. K. Pon , A. J. Gill , K. Moore , A. J. Spillane , et al. 2013 Breast cancer‐associated fibroblasts induce epithelial‐to‐mesenchymal transition in breast cancer cells. Endocr. Relat. Cancer 20:1–12. 23111755 10.1530/ERC-12-0227

